# Quick Rinse, Strong Bond? Comparing Short Water Rinsing and Advanced Cleaning Methods After Hydrofluoric Etching of Lithium Disilicate Glass Ceramic

**DOI:** 10.3390/ma19020299

**Published:** 2026-01-12

**Authors:** Viktoria Brandl, Matthias Kern, Maximiliane Amelie Schlenz, Sebastian Wille

**Affiliations:** Department of Prosthodontics, University Hospital Schleswig-Holstein, Campus Kiel, Christian Albrecht University of Kiel, Arnold-Heller-Str. 3, 24105 Kiel, Germanysebastian.wille@uksh.de (S.W.)

**Keywords:** lithium disilicate, cleaning methods, hydrofluoric acid, tensile bond strength

## Abstract

This study examined whether short water rinsing after hydrofluoric acid (HF) etching achieves comparable total bond strength (TBS) to more advanced cleaning protocols. Ninety-six lithium disilicate specimens were etched with 5% HF and then assigned to one of six post-etch cleaning methods: a 15 s water spray, 60 s water spray, brushing with a toothbrush, an ultrasonic bath with distilled water, an ultrasonic bath with 99% isopropanol, or a 37% phosphoric acid followed by an ultrasonic bath. The specimens were then bonded to acrylic tubes filled with composite resin. Half of the specimens were stored in water at 37 °C for three days, and the other half were stored for 150 days with 37,500 thermal cycles (5 °C/55 °C). TBS testing, failure mode evaluation, and microleakage testing were performed. Two-way ANOVA and Tukey’s test were used for statistical evaluation. Aging for 150 days significantly reduced TBS in all groups. Cleaning with a 60 s water spray resulted in significantly higher TBS than phosphoric acid plus ultrasonic cleaning, regardless of storage time. No significant differences were found among the other cleaning methods. There was no change in microleakage among the different groups; the failure was predominantly cohesive. A 15 s water spray after HF etching was as effective as more complex cleaning protocols in terms of TBS and SEM-observed surface characteristics.

## 1. Introduction

In clinical practice, adhesive luting of lithium disilicate restorations is often necessary due to their favorable mechanical properties and esthetic advantages [1,2,3,4,5]. In this context, the conditioning of the tooth and the restoration’s luting surface are both crucial. The adhesive tensile bond strength (TBS) to enamel is 30 MPa [6]. Sufficient bonding also reduces the occurrence of microleakage, which prevents visible discoloration at the margin. Microleakage allows bacteria to penetrate and weakens the adhesive bond through hydrolysis. To ensure optimal adhesion, the luting surface of lithium disilicate should be etched with 5% hydrofluoric acid (HF) after the try-in procedure. Manufacturers typically recommend a 5% HF etching time of 20 s to create a microstructure [3,7], expose bonding sites, and increase surface free energy [8,9,10]. HF etching exposes and generates hydroxyl groups on the ceramic surface that are responsible for chemical bonding.

After etching, fluoride salt residues from the crystalline phase remain on the surface and must be removed [11].

However, the literature presents controversial recommendations regarding the subsequent cleaning of the HF-etched lithium disilicate surface [11,12,13,14,15,16,17,18]. For instance, some studies report that removing the HF with a 60 s water spray, followed by a 5 min ultrasonic bath, yields the highest SBS results and a cleaner ceramic surface in a scanning electron microscope (SEM) analysis [12]. Other studies suggest applying a neutralizing agent, followed by ultrasonic cleaning [13], or cleaning in an ultrasonic bath alone would be sufficient [11,17,18]. It has also been claimed that an active application (agitated on the surface, and the load was calibrated in an analytical balance (up to 250 g)) of phosphoric acid might increase the SBS [14,19]. However, some studies have reported that different cleaning methods did not seem to affect the resin bonding [15,16]. In routine clinical practice, a simpler cleaning method may be advantageous, as it reduces errors, improves safety, and increases workflow. The more complex the cleaning protocol is, the higher the risk of procedural errors. These challenges led the authors to question whether a simplified approach—specifically, rinsing with water for 15 s—might be sufficient to achieve a reliable adhesive bonding.

The present study examined the TBS of lithium disilicate ceramic adhesive bonded with Variolink Esthetic DC after etching with HF and cleaning with different methods. Additionally, the microstructure and the appearance of the lithium disilicate ceramic surface and the microleakage were investigated after artificial aging. The first null hypothesis was that the cleaning methods would not affect the TBS between lithium disilicate and the resin composite-filled acrylglas tube and that cleaning with a 15 s water spray is sufficient, especially after artificial aging. The second null hypothesis was that cleaning methods would influence the lithium disilicate surface, leaving fluoride salt residues from the crystalline phase behind. The third hypothesis was that different cleaning methods would result in different levels of microleakage at the margins of the restorations.

## 2. Materials and Methods

### 2.1. Specimen Preparation and Test Groups

A total of 96 lithium disilicate specimens (IPS e.max Press, Ivoclar, Schaan, Liechtenstein) were fabricated by the manufacturer with a final diameter of 7 mm and height of 3.5 mm. Afterwards, the specimens were wet polished using a silicon carbide abrasive paper (320 grit and 600 grit) on a grinding machine (EcoMet 250 Pro, Buehler, Lake Bluff, IL, USA) for 20 s under a single contact pressure of 20 N. This polishing protocol resulted in a sufficient surface quality. The sample size of 96 was chosen based on previous studies in which a similar number of samples allowed statistically significant differences to be detected [20,21,22]. In this study, e.max press was used instead of e.max CAD/CAM because it offers cleaner and more precise marginal integrity for a restoration [23]. Pressed lithium disilicate restorations are less prone to chipping, and the marginal line and the surface are smoother. In addition, a higher level of detail is possible when modeling from wax. The flexural strength, fracture toughness, and Vickers hardness are higher. Lithium disilicate (Li_2_O·2SiO_2_) typically consists of 70% needle-shaped lithium disilicate crystals embedded in a glass-like matrix. The crystal size is approximately 3 to 6 µm [24].

First, all specimens were ultrasonically cleaned (Elmasonic S 30 H, Elma Schmidbauer, Singen, Germany) in 99% isopropanol (Otto Fischer, Saarbrucken, Germany) at a frequency of 40 kHz for 5 min. Afterwards, the specimens were dried with water- and oil-free air. Then specimens from all groups were etched with 5% HF (IPS Ceramic Etching-gel, Ivoclar) for 20 s and rinsed in a neutralization bath (1 measuring spoon per specimen powder mixture of sodium bicarbonate and calcium bicarbonate in a polyethylene cup (approx. 250 mL) with water) with water spray for 15 s (except for group W60 with 60 s). During application and removal of the hydrofluoric acid, the specimens were handled with rubber-coated tweezers while wearing protective gloves (latex) and protective glasses with a tight fit, as well as a face mask. Regarding the cleaning protocol of the surface, the specimens were divided into the following test groups (*n* = 16):

- No additional procedure, just the above-described cleaning with water spray for 15 s (W15).
- No additional procedure, just an extended cleaning time with water spray for 60 s (W60). Control group.
- Additional brushing with a disposable toothbrush (medium hard) without paste for 20 s (BRU).
- Additional ultrasonic bath with distilled water for 2 min (UBW).
- Additional ultrasonic bath with 99% isopropanol for 2 min (UBA).
- Additional cleaning with 37% phosphoric acid for 2 min followed by an ultrasonic bath with distilled water for 2 min (PHA + UBW).

After cleaning, all specimens were dried with water- and oil-free air.

Two specimens from each group were examined for differences in microstructure and surface roughness between the different cleaning methods. Therefore, the specimens were sputtered (SCD 500 Sputter Coaster, BAL-TEC, Wetter, Germany) with a layer of 10 nm gold and investigated in an SEM (Zeiss Supra 55 VP, Zeiss, Ulm, Germany) with an acceleration voltage of 10 kV.

In all groups, the lithium disilicate specimens were conditioned (according to the manufacturer’s instructions) by a microbrush with a thin layer of a freshly mixed two-component silane (Hoffmann’s Silan, Hoffman Dental Manufaktur, Berlin, Germany) for 30 s and dried with water- and oil-free air (visual control). The silane components A and B were mixed in a 1:1 ratio. Acrylic glass tubes with a length of 15.5 mm and an inner diameter of 3.2 mm (this dimension is required for fixation in the universal testing machine) were filled with self-curing luting resin composite, which was applied according to the manufacturer’s instructions (Clearfil Core New Bond, Kuraray Medical, Osaka, Japan). The resin composite was completely cured after 7 min. Using a custom-made bonding device [25,26,27], the acrylic glass tubes were bonded to the lithium disilicate ceramic specimens with a light- and dual-curing luting composite, Variolink Esthetic DC (Ivoclar). The bonding device had a defined contact pressure with a weight of 750 g; this weight corresponds to other studies [25,26,27] and enabled axial bonding. With a foam pellet, excess resin was removed, and glycerine gel (Liquid strip, Ivoclar, Schaan, Liechtenstein) was applied around the bonding margins to prevent oxygen inhibition. Polymerization was performed for 20 s using a light-curing lamp (Radii Cal, SDI, Bayswater, Victoria, Australia). Then, the specimens were placed in a light-curing oven (HiLite power, Heraeus, Hanau, Germany) for 180 s for complete polymerization. The glycerine gel was rinsed off with a water spray. Afterwards, the specimens were stored in a water bath at 37 °C in an incubator (Precision GP 02, Thermo Fischer Scientific, Freiburg, Germany). All materials used in this study are listed in Table 1.

### 2.2. Microleakage

Prior to TBS testing, all specimens were immersed in a 0.5% fuchsin solution (Merck, Darmstadt, Germany) for 24 h in an incubator, followed by cleaning under rinsing water. Subsequently, TBS testing, the penetration depth of the fuchsin solution into the bonding margin was evaluated using an optical microscope (Wild M420, Wild Heerbrugg, Heerbrugg, Switzerland) at 25× magnification.

### 2.3. Tensile Bond Strength Test

Test groups were split into two subgroups with 8 specimens in each group. One subgroup was stored for 3 days in 37 °C water to evaluate initial TBS, while the other subgroup was stored for 150 days in water with an additional 37,500 thermocycles between 5 °C and 55 °C as an aged group. Then, the tensile bond strength was measured using a universal testing machine (Zwick Z010/TN2A, Zwick Roell, Ulm, Germany) at a crosshead speed of 2 mm/min. Each specimen was mounted vertically into the testing machine using a chain loop arrangement that enabled torque-free axial clamping. The acrylic glass tube was fixed in the upper part with three clamping jaws, and the lithium disilicate specimen was fixed in the lower part in crescent-shaped recesses on both sides [22,28,29]. An axial load was applied to the adhesive joint of the specimens by tensioning the chain loops in the upper and lower parts. Using testing software (TestXpert II (V.3.71), Zwick Roell), the tensile bond strength was calculated in MPa by dividing the maximum load at failure by the bonding area (8.04 mm^2^). The TBS data were statistically analyzed using two-way ANOVA and the post hoc Tukey test. The normal distribution of the data was previously analyzed using the Shapiro–Wilk test and was not disproved (*p* > 0.05).

### 2.4. Failure Modes

Failure modes were examined using an optical microscope at 25× magnification. The failure modes of each specimen were recorded as a percentage and classified into the following categories:

- Adhesive failure (A): debonding at the lithium disilicate ceramic surface.
- Cohesive failure (C): failure within the luting resin composite or within the acrylic glass tube-filling resin composite.

In addition, two representative specimens from each group were analyzed in an SEM (XL 30 CP, Philips, Kassel, Germany) on the failure mode (65× and 100× magnification).

## 3. Results

**Figure 1:**
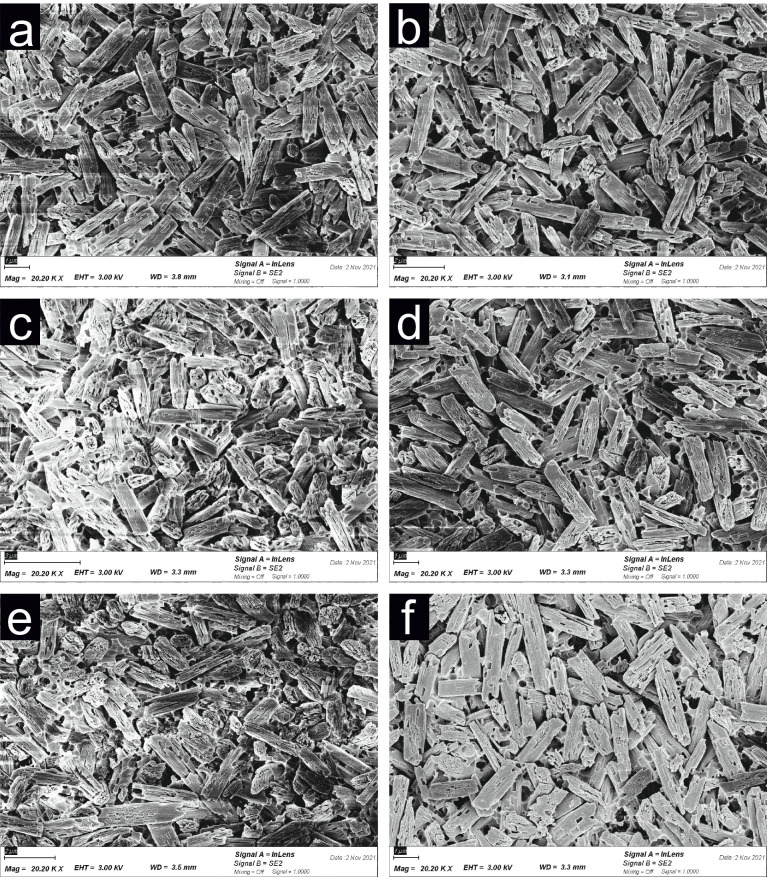
Scanning electron micrograph images (20,200× magnification) of the lithium disilicate ceramic surface after HF etching and cleaning in the different test groups (**a**): W15 = No additional procedure, just the described above cleaning with water spray for 15 s, (**b**): W60 = No additional procedure, just an extended cleaning time with water spray for 60 s, (**c**): BRU = Additional brushing with a disposable toothbrush without paste for 20 s, (**d**): UBW = Additional ultrasonic bath with distilled water for 2 min, (**e**): UBA = Additional ultrasonic bath with isopropanol 99% for 2 min, (**f**): PHA + UBW = Additional cleaning with 37% phosphoric acid for 2 min followed by ultrasonic bath with distilled water for 2 min). Every group showed only lithium disilicate needles on the surface.

The evaluation of the SEM images showed no significant difference between the test groups (Figure 1). Additional cleaning procedures did not change the microstructure or the surface appearance of the lithium disilicate ceramic, and no residues of HF or the silicate fluorine salt were detected on the evaluated specimens.

The mean TBS values after 3 days of water storage and after 150 days with thermocycling are presented in Table 2. There was no interaction between cleaning methods and storage time (*p* = 0.427). Thermocycling for 150 days caused a statistically significant decrease in TBS for all test groups compared to the initial bond strength after 3 days of water storage (*p* < 0.001).

Cleaning with water spray for 60 s (group W60) showed statistically significant higher TBS compared to cleaning with phosphoric acid followed by ultrasonic cleaning in distilled water (group PHA + UBW) under both storage conditions (*p* = 0.035). No statistically significant differences could be revealed (*p* > 0.05) between the other cleaning methods.

**Figure 2:**
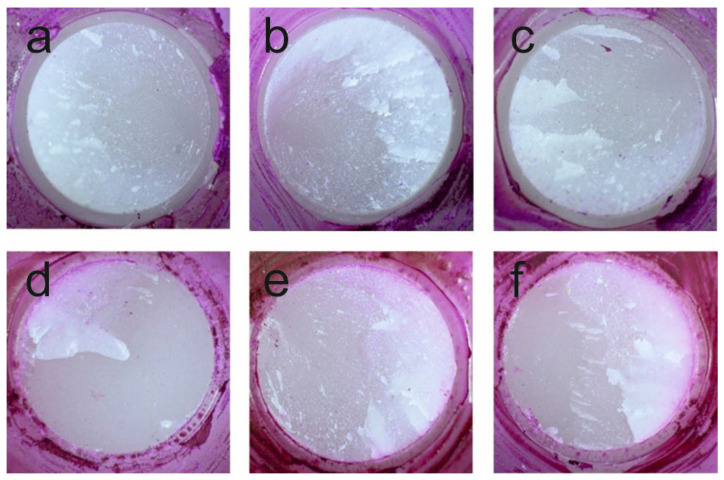
(**a**): W15 = No additional procedure, just the above-described cleaning with water spray for 15 s; (**b**): W60 = No additional procedure, just an extended cleaning time with water spray for 60 s; (**c**): BRU = Additional brushing with a disposable toothbrush without paste for 20 s; (**d**): UBW = Additional ultrasonic bath with distilled water for 2 min; (**e**): UBA = Additional ultrasonic bath with 99% isopropanol for 2 min; (**f**): PHA + UBW = Additional cleaning with 37% phosphoric acid for 2 min followed by ultrasonic bath with distilled water for 2 min. Example for the penetration of the fuchsin solution under the microscope (25× magnification). No penetration on the bonding area and no difference between the different cleaning groups were evaluated. The penetration depth of the fuchsin solution did not extend to the edge of the acrylic glass tubes. Therefore, no penetration in the bonding area and no difference between the different cleaning groups were observed.

The microscopic examination of microleakage at the bonding margin with fuchsin solution showed minor microleakage at the interface between the lithium disilicate and the acrylic glass tube, but no penetration was observed on the bonded lithium disilicate surface for any of the tested cleaning methods (Figure 2).

**Figure 3:**
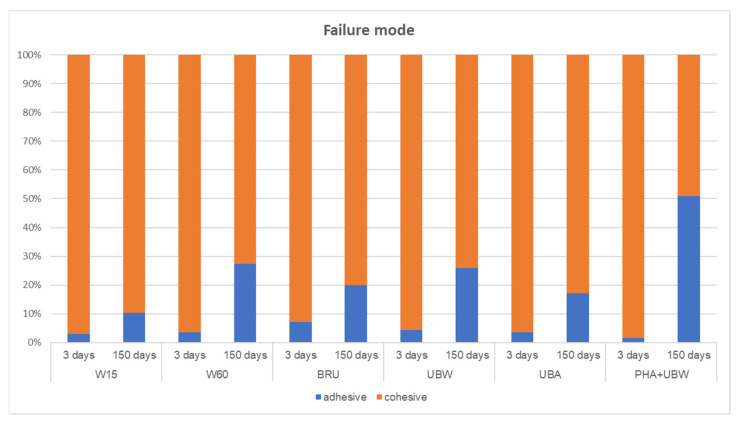
Mean percentages of the failure modes in test groups (W15 = no additional procedure, just the above-described cleaning with water spray for 15 s; W60 = no additional procedure, just an extended cleaning time with water spray for 60 s; BRU = additional brushing with a disposable toothbrush without paste for 20 s; UBW = additional ultrasonic bath with distilled water for 2 min; UBA = additional ultrasonic bath with 99% isopropanol for 2 min; PHA + UBW = additional cleaning with 37% phosphoric acid for 2 min followed by ultrasonic bath with distilled water for 2 min) after tensile bond strength testing after 3 and 150 days of storage. Adhesive failure (blue): failure at the lithium disilicate ceramic surface. Cohesive failure (orange): debonded failure in the luting resin composite or in the acrylic glass tube-filling resin composite. No damage to the lithium disilicate surface was observed. The failure mode was mostly cohesive in the resin in all groups, but the portion of adhesive failure increased in all groups after 150 days of water storage with thermocycling.

The failure mode in all groups was mostly cohesive in the resin; however, the portion of adhesive failure increased in all groups after 150 days of water storage with thermocycling (Figure 3). A higher ratio of adhesive failure mode indicates a lower adhesive bond to the lithium disilicate ceramic. This is an indication of residue on the adhesive surface after etching, meaning that the cleaning method is insufficient. Aging induces hydrolytic degradation of the adhesive resin, thereby weakening the adhesive bond. Differences in thermal expansion coefficients may further compromise the bond due to temperature cycling between 5 and 55 °C. Consequently, a higher failure rate is expected in samples from the 150-day series. The increased proportion of adhesive failure observed after 150 days suggests a greater risk of complications with prosthetic restorations in the oral environment after approximately 4–7 years.

**Figure 4:**
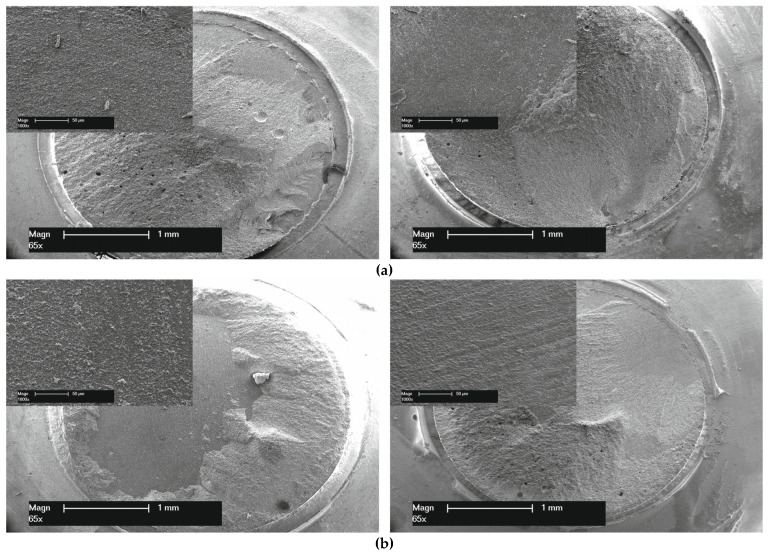
Scanning electron micrograph images (65× and 100× magnification) showing an example of the failure modes of the test group. No additional procedure, just the described above cleaning with water spray for 15 s (W15) with a detailed image (100× magnification) in the upper left corner, illustrating the presumed adhesive fracture region, which remains partially covered by a thin layer of luting composite after 3 days ((**a**), **left**) and 150-days storage ((**a**), **right**) and the test group “Additional cleaning with 37% phosphoric acid for 2 min followed by ultrasonic bath with distilled water for 2 min (PHA + UBW) with a detailed image (100× magnification) in the upper left corner from 3 Days storage, which demonstrates that the presumed adhesive fracture area is still slightly covered with a layer of luting composite ((**b**), **left**). The detailed image from 150 days of storage displays the clean, polished surface of the lithium disilicate ceramic ((**b**), **right**).

The evaluation of the SEM images after the TBS test showed both an adhesive and a cohesive failure mode. Most of the failures were cohesive fractures (<50%) within the luting resin composite or the acrylic glass tube-filling resin composite. Exemplary samples from each group are displayed in Figure 4.

## 4. Discussion

The mean TBS of all test groups exhibited a bond strength of more than 30 MPa, which corresponds to the adhesive tensile bond strength to enamel [6]. This contradicts the results of the studies that HF etching should be followed by a 5-min cleaning in an ultrasonic bath [12] and that a neutralizing agent should be applied, followed by ultrasonic cleaning [13]. It also contradicts the studies that cleaning in an ultrasonic bath alone is sufficient [11,18] and that an active application of phosphoric acid increases the bond strength to lithium disilicate ceramic [14,19]. In other studies [11,12,13,14,15,16,17,18], different bonding agents and silanes were used, which may also be a reason for the varying results. However, other studies [16,17] are consistent with the results of this study that cleaning with a water spray is effective. The SEM evaluation showed no differences in the appearance of the lithium disilicate ceramic surface after using the different cleaning protocols.

Artificial aging (150 days with thermocycling) caused a statistically significant decrease in TBS in all groups, which is consistent with the literature [21,25,30,31,32]. Cleaning with water spray for 60 s (group W60) resulted in a significantly higher TBS than cleaning with phosphoric acid followed by ultrasonic cleaning in distilled water (group PHA + UBW), independent of the storage conditions. No statistically significant differences were observed between the other cleaning methods. According to the first null hypothesis, that the cleaning methods would not affect the TBS and that cleaning with a 15 s water spray is sufficient, particularly after 150 days of thermocycling, must be partially rejected.

All cleaning methods resulted in microleakage-free resin bonding (only the bonding margin showed minor microleakage at the interface between the lithium disilicate and the acrylic glass tube) to lithium disilicate ceramics, which also corresponds to the high TBS values after 3 and 150 days of water storage. Therefore, the second hypothesis that the cleaning methods influence the lithium disilicate surface and the microleakage at the margin of the restorations, which was analyzed with a fuchsin solution, must be rejected as well.

The high TBS might also be related to the specific silane used in this study, which was freshly mixed and activated immediately before application. This mixture produces a highly reactive silane that does not have to compete with other monomers for binding sites, as in so-called universal primers [21,25]. Silanes have a bifunctional structure and are used for the chemical cross-linking between ceramic particles and adhesive particles.

Artificial aging caused a statistically significant decrease in TBS in all groups, which indicates an aging process of the adhesive materials [20]. Even after long-term aging (150 days with thermocycling), which is equivalent to 4–7 years of clinical aging [33], the adhesive bond of all test groups exhibited a TBS of over 30 MPa. This TBS corresponds to the adhesive tensile strength of enamel [6]. The results indicate that a simple cleaning approach involving a 15 s water spray after HF etching appears sufficient for achieving durable bonding to lithium disilicate ceramic.

E.max Press has an extended microcrystal; they are longer compared with the crystals in the e.max CAD version. These longer crystals produce a stronger final restoration. The various crystal morphologies may influence etching behavior, but no significant changes are to be expected. It is quite unlikely that there will be a different outcome of the study results by using the e.max CAD version instead of the e.max Press.

A limitation of this study is the use of specimens with flat surfaces. Further studies, particularly clinical investigations involving teeth and crowns with irregular surfaces and lumens that increase the complexity of the cleaning process, are highly recommended. It should be noted that HF etching raises several health, safety, and environmental concerns due to the toxicity of the acid. The etching process may be safer in a dental laboratory, where appropriate safety equipment is available, than in a clinical setting close to the patient.

## 5. Conclusions

Within the limitations of this in vitro study, a 15 s water spray cleaning following HF etching of lithium disilicate ceramic proved to be as effective as more advanced cleaning protocols without a clinically significant decrease in tensile bond strength. Staining with fuchsin solution also showed no differences in microleakage between the groups, and the failure mode was, in all groups, mostly cohesive.

## Figures and Tables

**Table 1:** List of materials used in this study.

Material	Manufacturer	Composition	Batch No.
IPS e.max press	Ivoclar, Schaan, Liechtenstein	Lithium disilicate ceramic (SiO_2_ 57–80%, Li_2_O 11–19%, K_2_O 0–13%, P_2_O_5_ 0–11%, ZrO_2_ 0–8%, ZnO 0–8%, other oxides and ceramic pigments 0–10%)	H21370
Clearfil Core New Bond	Kuraray Medical, Osaka, Japan	Bisphenol A diglycidylmethacrylate (Bis-GMA), triethyleneglycol dimethacrylate (TEGDMA), silanated glass filler, colloidal silica, catalysts, accelerators	000148
Variolink esthetic DC	Ivoclar, Schaan, Liechtenstein	Urethane dimethacrylate, further methacrylate monomers, inorganic fillers 38% (ytterbium trifluoride, spheroid mixed oxide), initiators, stabilizers, pigments, additional ingredients	Z01579
Hofmann’s Silan	Hoffman Dental Manufaktur, Berlin, Germany	Acetic acid in ethanol/water mixture, 3-methacryloxypropyl-trimethoxysilane in ethanol/water mixture	8821/9078
Liquid strip	Ivoclar, Schaan, Liechtenstein	>90% glycerine, silicon dioxide and alumina	Z017TO
Pluraetch	First Scientific Dental Materials, Elmshorn, Germany	37% phosphoric acid on thixotropic gel base	52003306
IPS Ceramic Etching-gel	Ivoclar, Schaan, Liechtenstein	5% hydrofluoric acid	Z015FK

**Table 2:** Mean and standard deviation (SD) of tensile bond strength [MPa] of the six test groups (n = 8). TC = thermocycling, W15 = no additional procedure, just the above-described cleaning with water spray for 15 s, W60 = no additional procedure, just an extended cleaning time with water spray for 60 s, BRU = additional brushing with a disposable toothbrush without paste for 20 s, UBW = additional ultrasonic bath with distilled water for 2 min, UBA = additional ultrasonic bath with 99% isopropanol for 2 min, PHA + UBW = additional cleaning with 37% phosphoric acid for 2 min followed by ultrasonic bath with distilled water for 2 min. Statistically significant different means (*p* ≤ 0.05) are indicated by different subscript lowercase letters (within a row, comparing 3- and 150-day storage within the same test group) or by superscript uppercase letters (within a column, for the same storage conditions).

Group	Storage Time			
	3 Days Without TC		150 Days/37,500 TC	
	Mean	SD	Mean	SD
W15	48.0_a_^AB^	13.3	42.2_b_^AB^	19.4
W60	49.7_a_^A^	13.8	42.5_b_^A^	19.5
BRU	47.0_a_^AB^	15.3	42.6_b_^AB^	13.4
UBW	48.6_a_^AB^	17.4	37.0_b_^AB^	30.6
UBA	47.7_a_^AB^	7.1	33.9_b_^AB^	23.7
PHA + UBW	40.6_a_^B^	18.1	34.9_b_^B^	24.5

## Data Availability

The original contributions presented in this study are included in the article. Further inquiries can be directed to the corresponding author.
